# Ether in the Passage of Gravel

**Published:** 1847-05

**Authors:** 


*Ether in the Passage of Gravel.*—(Communicated by Dr. Ware, to the Boston Society of Medical Improvement.)—The inhalation of sulphuric ether was recently employed, in this city, for the relief of the pain attendant on the passage of a renal calculus through the ureter, in a case under the care of Drs. Homans and Ware. The patient, a gentleman of about 50 years of age, was convalescent from pleuro-pneumonia, having still some pain in the side, cough and bloody expectoration. About 7 o'clock in the evening, immediately after an evacuation from the bowels, he was seized with intense pain, in a spot on the left side, midway between the umbilicus and the crest of the ileum. Elixir of opium in the course of an hour and a half, was given to the amount of 400 drops; several enemata were administered, and various local applications were made, with but slight alleviation, and the suffering was still of the most severe description.

At a quarter before nine, the ether was administered by Dr. Keep. The patient did not become at any time entirely insensible, but was very soon comparatively easy, and remained so as long as the influence of the remedy continued. As soon as the pain returned, he requested a repetition of the inhalation, and was again relieved. The pain still returned, from time to time, as the influence of the ether subsided, but was kept under by a renewal of the process until three o'clock in the morning, when the patient became so easy, that it was no longer necessary. During this period, of about six hours, there were thirty repetitions of the operation which were counted, besides several others which were not.

There was no subsequent return of the pain, and no inconvenience whatever was experienced. The pain in the side and bloody expectoration almost immediately ceased; and the patient states, that a pain to which he has been subject for many years, when lying on the left side, has left him since the inhalation.

The following practical inferences seem worthy of being noticed in connection with this case.

1. That a pain of this description, which is not relieved by large doses of opium, may be mitigated by inhalation of ether, without suspending that natural course of things by which its cause will be sooner or later removed. Hence this remedy may be applicable during the passage of a biliary calculus, in colic or other cases of spasm, and, perhaps in some cases of even natural labor.

2. That an individual may be kept under the continued influence of ether, for a long period of time with safety.

3. That the inhalation is not necessarily irritating to even diseased lungs, and the presence of considerable disease in them does not constitute an objection to its use, when circumstances demand it.

The practical bearing of these inferences is not affected by any doubts as to the accurcy of the diagnosis. Even were the medical attendants wrong in their jndgment on this point, which is very possible, it is not the less true that the patient was speedily and for a long time relieved of intense suffering, that the cause of the pain was during this time removed, and that no subsequent ill effects were the result. *Boston Medical Journal.*

